# Decision Support Tool to Improve Decision-Making for HIV Pre-Exposure Prophylaxis (PrEP): Development Process and Alpha Testing

**DOI:** 10.2196/57348

**Published:** 2024-10-11

**Authors:** Wale Ajiboye, Abban Yusuf, Cheryl Pedersen, Rebecca Brown, Kristaps Dzonsons, LaRon Nelson

**Affiliations:** 1 MAP Center for Urban Health Solution St. Michael's Hospital Unity Health Toronto Toronto, ON Canada; 2 Tuliptree LLC Dover, DE United States; 3 Yale School of Nursing Yale University New Haven, CT United States

**Keywords:** HIV PrEP in black patients, pre-exposure prophylaxis, decision support tool to increase PrEP uptake and adherence, HIV prevention in Black communities

## Abstract

**Background:**

African, Caribbean, and Black (Black) communities in Canada are disproportionately affected by the HIV epidemic. Pre-exposure prophylaxis (PrEP) is a highly effective option for the prevention of HIV. However, the use of PrEP for HIV prevention among eligible Black clients in Canada remains far below the thresholds necessary to achieve the goal of zero new HIV infections. In a recent study in Toronto, PrEP-eligible Black clients were found to have decisional conflict and unmet decisional needs, which affected the quality of their decision-making process regarding the initiation and adherence to PrEP. There is evidence that decision support tools (DSTs) can improve the quality of a decision, the quality of the decision-making process, the implementation or continuation of the chosen option, and the appropriate use of health services. Despite these benefits, there is currently no DST for PrEP-eligible Black clients being asked to consider PrEP for HIV prevention.

**Objective:**

Our study aimed to develop a DST to improve PrEP decision-making for Black clients and to evaluate the tool’s acceptability and usability.

**Methods:**

We developed and evaluated the PrEP DST for Black patients using the 7-step process outlined in the Ottawa Decision Support Group Guideline for the development and evaluation of DST. To facilitate the implementation of the Ottawa Decision Support Group guideline, we assembled a multidisciplinary team of primary health care providers, researchers, community members with lived experiences, and digital content designers to serve as the steering committee. First, we assessed patients’ and primary health care providers’ views on decisional support needs, after which we determined the content, design, and distribution plan for the DST. Subsequently, we conducted evidence synthesis, reviews, and appraisal before developing the PrEP DST prototype. The final tool was reviewed by steering committee members for completeness before acceptability and usability testing with potential Black clients and PrEP providers.

**Results:**

The web-based DST yielded 27 pages divided into 6 distinct sections. The six sections include (1) an introduction of the DST, (2) clarify your decision, (3) knowledge, (4) a value clarification exercise, (5) support system, and (6) next steps. Both Black clients and PrEP providers reported ease of task performance, general satisfaction, and usefulness of the tool to support decision-making for Black clients. Feedback on usability centered on the need to add a user guide to increase usability. All feedback was incorporated into the final tool.

**Conclusions:**

A PrEP DST for Black clients developed using a systematic process and a multidisciplinary steering committee was acceptable and usable by both Black clients and PrEP providers. Further study (eg, randomized controlled trials) may be needed to evaluate the efficacy of the PrEP DST.

## Introduction

### Background

African, Caribbean, and Black (Black) communities in Canada are disproportionately affected by the HIV epidemic [1]. Despite making up 4.3% of the population, Black communities in Ontario accounted for 25% of new HIV infections in 2021 [1,2]. In a recent cross-sectional survey of Black people in Ontario aged 15-64 years, HIV prevalence was found to be 7.5% [3]. These data are concerning and provide a basis to develop and implement strategies to increase the acceptance and use of effective tools for HIV prevention in Black communities in Canada. There are multiple options to prevent HIV infection—abstinence, never sharing needles, using a condom the right way, HIV pre-exposure prophylaxis (PrEP), HIV post exposure prophylaxis, and HIV treatment as prevention [4]. Among these options, PrEP is the most commonly prescribed HIV prevention option for eligible Black Canadians [5]. PrEP is an effective HIV prevention medication that is recommended for communities with high ongoing risks of HIV infection [6,7]. However, acceptance of PrEP among Black Canadians is low [5]. Factors affecting acceptance of PrEP are multidimensional and are related to lack of knowledge, low perception of risk of HIV acquisition, preference for other prevention options, health care providers’ attitudes, cost of PrEP, lack of drug coverage, lack of a primary care physician, inadequate consultation time with primary health care provider, anti-Black racism, and PrEP stigma [8-13]. In addition, for most PrEP-eligible Black Canadians, the availability of multiple options to prevent HIV, and the lack of adequate knowledge about these options, including information on the benefits and drawbacks of these options present decision-making challenges on whether to accept PrEP or not for HIV prevention [12]. These challenges underscore the necessity to improve the decision-making process for Black Canadians who are being asked to consider PrEP for HIV prevention.

There is evidence that decision support tools (DSTs) can improve the decision-making process for individuals facing health care decisions [14,15]. A systematic review of the literature on DST concluded that DSTs can increase participants’ knowledge, increase the accuracy of risk perceptions, and the congruency between informed values and care choices [14]. It also improves both the quality of the decision process and the decision itself. Decision support interventions involve (1) establishing rapport and facilitating interactive communication, (2) clarifying the decision and inviting patient participation, (3) assessing the patient’s decisional needs, and (4) addressing decisional needs with tailored support. Decision support interventions may be administered using various media such as web-based tools, mobile apps, decision boards, interactive videodiscs, personal computers, audio-guided workbooks, pamphlets, and group presentations [16]. Decision support interventions have been developed for various medical therapies, diagnostic tests, preventive therapies, clinical trial entry decisions, and end-of-life decisions [14,17]. However, there is currently no patient decision-support intervention for Black patients being asked to consider PrEP for HIV prevention, especially in the context of previous studies indicating that most of these patients do have decisional conflict regarding PrEP [12,17]. Therefore, this study aimed to develop and evaluate a decision-support intervention to improve the decision-making process for Black patients who are offered PrEP for HIV prevention.

### Theoretical Frameworks

**Figure 1:**
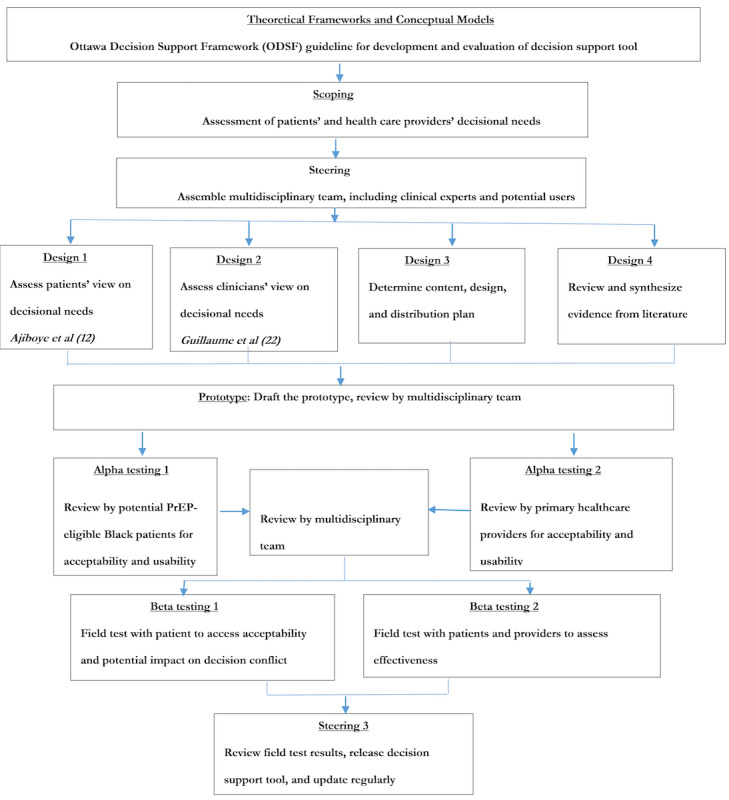
Schematic presentation of the development process and evaluation of HIV pre-exposure prophylaxis Decision Support Tool [12,22]. PrEP: pre-exposure prophylaxis.

The guideline for Developing and Evaluating Decision Support Intervention developed by the Ottawa Decision Support Group was used to guide the development and evaluation of the PrEP DST [18]. The guideline recommends 7 steps in developing and evaluating a decision aid [13]. These steps include (1) assessing needs, (2) assessing feasibility, (3) defining the objectives of the aids, (4) identifying the framework of decision support, (5) selecting the methods of decision support to be used in the aid, (6) selecting the designs and measures to evaluate the aid, and (7) planning dissemination. In step 4, we selected the Ottawa Decision Support Framework as the framework for the PrEP decision support [19]. In step 5, we used the Ottawa Personal Decision Guide (OPDG) to determine the content and interconnectivity of the various sections of the DST [20]. The reporting guideline recommended by Coulter et al [21] was used to guide the reporting of our DST as shown in Figure 1 [12,22]. Coulter et al [21] recommended 5 themes for documenting the development process of DST: (1) scoping (the assessment of decisional needs of patients and primary health care providers), (2) steering (engagement of multidisciplinary team, and the process used to determine content, design, and distribution plan), (3) design (how evidence included in the decision support were identified, synthesized, reviewed, and appraised, and the development of the prototype), (4) alpha testing (review by potential users for acceptability and usability), and (5) beta testing (pilot randomized controlled trial).

## Methods

### Ethical Considerations

This study’s approval was obtained from the Research Ethics Board of St. Michael Hospital, Unity Health Toronto (#18-022). Verbal consent was obtained from all study participants following a detailed explanation of this study’s purpose and procedure. Oral and written consent were sought and received before this study. All study data were deidentified and stored in the hospital’s secured server.

### Study Setting and Recruitment Procedure

This study was conducted at MAP Center for Urban Health Solution. MAP is based at St. Michael’s Hospital, a fully affiliated University of Toronto teaching and research-intensive hospital. The hospital is also a hub for care in downtown Toronto.

Study participants were recruited through St. Michael’s Hospital Family Health Teams, and PrEP providers in downtown Toronto.

### Scoping: Assessment of Patients’ and Primary Health Care Providers’ Views on Decisional Need

We conducted key informant interviews of 29 PrEP-eligible Black clients of different genders, sexual identity, and at different decision-making stages to understand their decision-making process and to assess their decision-support needs for PrEP. The result of this phase of the development process has been reported by Ajiboye et al [12]; the study includes an explanation of who identified participant race and ethnicity and is also the source of the classifications used. We also surveyed 25 PrEP providers (clinical and primary health care service providers) to assess needs and preferences for a DST for PrEP-eligible Black patients. The result of the PrEP-providers’ survey has been published by Guillaume et al [22]. Data obtained from the patient’s interview and primary health care providers’ survey were used to inform the content, design, and distribution plan for the PrEP DST.

### Steering: Determine Content, Design, and Distribution Plan

To guide the development of the PrEP DST, we set up a multidisciplinary team comprising of researchers, primary health care providers, PrEP-eligible Black patients, HIV service providers, and information technology experts. Participants were selected based on their experience in HIV prevention in Black communities, provision of decision support to PrEP-eligible Black patients, and knowledge of digital health technology design. The team met regularly (weekly initially and then monthly subsequently) to determine the content, design, and distribution plan for the tool. Additionally, we also met as a team or in pairs to identify, synthesize, review, and appraise evidence for inclusion in the tool as well as to review the draft prototype and the results of the alpha testing of the tool. For the content of the tool, we used the OPDG to determine the contents and the interconnectivity of the different sections of the tool. The design and the distribution plan were informed by the goal of the DST and the information obtained from the patients’ and primary health care providers’ assessment of decisional needs.

### Design: Evidence Synthesis, Review and Appraisal, and Development of Prototype

#### Evidence Synthesis, Review, and Appraisal

We used several sources to identify evidence for inclusion in the DST. A search of both published and grey literature was conducted [4,6,23-33]. We also reviewed data obtained from our study of patients’ and primary health care providers’ assessment of decisional needs [12,22]. Data from our study on the assessment of patients’ decision support needs and preferences were qualitatively analyzed. A content analysis technique was used to identify words, themes, and concepts related to the ODPG. Further, 2 steering committee members (LN and WA) reviewed the content separately before the final review by the steering committee. We only included words, themes, and concepts in which the committee achieved a high level of agreement. Overall, all the evidence synthesized from the literature was appraised and summarized. Where available, we prioritized evidence from meta-analyses, systematic reviews, randomized control trials, and cohort or case-control studies for inclusion in the tool. Evidence from the websites of scientific organizations was screened for relevance before inclusion in the tool.

#### Development of the Prototype

The prototype was developed using the drafting and redrafting process. PC, RB, and WA wrote the first draft of the script using evidence synthesized from the literature, and results from our study of patients' and primary care providers’ decision needs, and websites of scientific organizations. LN reviewed it for completeness and compliance with the OPDG. Subsequently, the multidisciplinary team reviewed the final prototype using specific criteria such as feasibility, appropriateness, and acceptability to assess the content, design, and decision support method. The final design was approved by the team after a thorough review of the content, flow, and design.

### Alpha Testing: Review by Potential Users for Acceptability and Usability

We recruited potential users—3 PrEP-eligible clients and 2 PrEP providers to review the DST for acceptability and usability. A link to the web-based DST was sent to all the reviewers so they could view the tool on their own devices and provide feedback on the following metrics: task performance (ease of navigating the tool and completion of all the tasks in the tool), and user satisfaction. Written feedback received from the potential users was reviewed by the multidisciplinary team, and useful suggestions were incorporated to improve the tool.

## Results

### Scoping: Assessment of Patients’ and Primary Health Care Providers’ Views on Decisional Needs

The result of the assessment of the decisional needs shows that most Black patients experience some form of decisional conflict—uncertainty on whether to accept PrEP for HIV prevention or to use other HIV prevention methods—when offered PrEP for HIV prevention. We also found that these uncertainties were due to a lack of adequate information on PrEP, including how it compares to other prevention methods for benefits and risks. In addition, most participants indicated a preference for a web-based or digital DST. Based on these results, we determined that the DST will aim to support Black patients to make evidence-based informed decisions on whether to use PrEP or not. In addition, we also determined that the DST will not seek to promote the use of PrEP for HIV prevention but will support Black patients to make informed and value-based decisions on HIV prevention methods. The goal is to reduce decision conflict for Black patients, promote evidence-based decisions on PrEP, and enhance implementation of the chosen HIV prevention option.

For the primary health care providers’ survey, we found that most primary health care providers (93%) agreed that a DST would complement the service they provide to their PrEP-eligible Black patients [12]. They identified Black patients’ need for culturally appropriate information on the benefits and risks of PrEP as one of the reasons why a DST would be needed. Most primary health care providers (95%) also agreed that a DST would facilitate the conversation on HIV prevention with Black clients [22].

### Steering: Content, Design, and Distribution Plan

Using the OPDG, we identified 6 content sections for the PrEP DST [34]. The six sections include (1) an introduction of the DST, (2) a clarify your decision section, (3) a knowledge section, (4) a value clarification exercise section, (5) a support system section, and (6) next steps. In order to enhance readability and comprehensibility, we used simple words and short sentences, avoided medical jargon, and included a plain language description of any medical term. We adopted a design that includes a step-by-step way of moving through the content of the tool to enhance readability.

For the distribution plan, we agreed to present the DST as a web-based tool that can be used for decision-making before or after visit to PrEP provider. For easy access, we determined it will be good to make it publicly available.

### Design: Evidence Synthesis, Review, and Appraisal, and Development of Prototype

#### Evidence Synthesis, Review, and Appraisal

Clinical evidence on probabilities of outcomes was difficult to find, and where available the evidence was generally weak and sometimes conflicting. Only outcomes obtained from evidence with strong methodology were included in the knowledge section of the DST. Data on the features of various HIV prevention options including the positive and negative features were primarily obtained from the decision needs assessment study, although we supplemented it with evidence from the literature where necessary. This is to ensure that the information included in the DST was all relevant to our target audience—PrEP-eligible Black clients.

#### Development of the Prototype

The prototype development resulted in a tool with 6 different sections. Section 1 contains a brief introduction to the DST including the objectives and instructions on how to log-in to access other sections of the tool. In section 2, users can clarify the decision being made and the reason for making the decision. Section 3 contains information on the various HIV prevention options and the positive and negative features of each of the options. We provided additional information on PrEP because this is the most recommended HIV prevention option, which is not well known to most Black clients. If available, we included the probabilities of outcomes of the different options in the information subsection or in the negative or positive features. Section 4 provided a method for users to select and rate the features of HIV prevention options that matter most to them. The outcome of the rating is presented in a visualized form so that users can see how the option selected matches their values. In section 5, users can identify and select their support system—individuals who can provide support for the implementation of the decision. Section 6 contains steps and actions the user needs to take to implement the decision made, including how to discuss their decision with their primary health care providers or others identified in their support system in section 5.

### Alpha Testing: Review by Potential Users for Acceptability and Usability

All the reviewers reported ease of task performance for all the sections of the DST. Additional comments on task performance centered on the provision of more information to guide the user in the performance of the various tasks in the tool. One of the participants expressed it this way: “can a progress bar be added so that users know how they are progressing in the tasks?”

All reviewers expressed general satisfaction and usefulness of the tool, although the issue of who may likely benefit the most from the tool was also raised. One of the participants expressed it this way:

I just went through the tool. Overall, I think it provides useful information and resources to help people decide whether or not PrEP is a good decision for them. I do, however, think that it skews toward people who are not yet taking PrEP. I went through the tool as someone who was considering taking PrEP and someone who was debating coming off PrEP just to see if the questions would be different and based on both walkthroughs, I think it’s less useful/applicable for someone who is in the latter category.

Another reviewer expressed satisfaction with the tool this way: *“*Cool! I checked the whole thing out. Very comprehensive. Great work. Thank you for sending.”

## Discussion

### Statement of Principal Findings

Our study developed a DST to help Black clients decide whether PrEP is a good HIV prevention option. Overall, this study produced a DST that is acceptable and usable for decision-making by PrEP-eligible Black clients and PrEP providers. To the best of our knowledge, our study is the first attempt to characterize Black clients’ decision-support needs regarding HIV prevention and to use this information to systematically develop a DST to enhance their decision-making for HIV prevention options [16,17].

### Strengths and Weaknesses of This Study

Several aspects of this study affirm the rigor of the methodology used for the development of the DST. The use of evidence-based frameworks (Ottawa Decision Support Framework and OPDG) to guide the process of development and alpha testing of the tool conforms to the recommendations of International Patient Decision Aid Standards, which states that patient DSTs should be carefully developed, user-tested, and open to scrutiny, with a well-documented and systematically applied development process [35,36]. In addition, our study used a qualitative approach to evaluate the acceptability and usability of the DST. This qualitative approach allows users the freedom to express their pain points freely when using the tool but does not give the benefits of quantifying users’ responses [37]. The use of mixed methods would have been ideal, but we were constrained by limited resources and time. However, the qualitative approach provided useful feedback that was incorporated into the final version of the DST. One of the major weaknesses of this study is the inability to compare the probabilities of outcomes for the various HIV prevention options. The evidence was not available in the literature; hence, we were limited to including what was available. We hope to update the tool once this information is available.

### Strength and Weakness Concerning Other Studies Particularly Discussing Any Differences in Results

The development process and testing of our DST compared favorably with other studies [14,16,21]. Coulter et al [21] noted that key features common to all DSTs’ development processes include scoping, development of a prototype; alpha testing with patients and primary health care providers in an iterative process; beta testing in real-life conditions (field tests); and production of a final version for use or further evaluation. The use of the evidence-based framework for the development process made it possible to capture all these key features in our study. In addition, most developers did not describe a distribution strategy for their DST; however, our study identified and described the distribution strategy for the DST during the planning phase [21].

### Meaning of This Study: Possible Mechanisms and Implications for Primary Health Care Providers or Policy Makers

This study highlights the benefits of using evidence-based frameworks and a multidisciplinary team to guide the development process of a DST. Second, the difficulty of comparing probabilities of outcomes in the light of limited evidence can be a major challenge. This challenge can, however, be minimized by regularly updating the DST once additional evidence becomes available. PrEP providers (clinical or primary health care service providers) now have an evidence-based tool that can be used to guide PrEP-eligible clients in making informed decisions about whether to use PrEP or not.

### Unanswered Questions and Future Research

Additional studies will be needed to assess the feasibility of implementing the DST in real life—beta testing. Second, an effectiveness trial will also be needed to ascertain the impact of the DST on decision-making, clinical outcomes, and use of the health care system.

### Conclusion

A DST was developed for PrEP-eligible Black patients to enhance their decision-making process for HIV prevention options. Potential users (Black patients and primary health care providers) found it usable. Additional studies will be needed to evaluate the feasibility of implementing the DST in a real-life setting [34].

## Abbreviations

DST: Decision Support Tool
OPDG: Ottawa Personal Decision Guide
PrEP: pre-exposure prophylaxis

## Data Availability

The web-based PrEP DST has been reviewed and approved by International Patient Decision Aid Standards for public use [38].

## References

[1] Ontario HIV Epidemiology and Surveillance Initiative (2022). A snapshot of HIV diagnoses and the HIV care cascade among African, Caribbean and Black people in Ontario.

[2] Public Health Agency of Canada (2022). HIV in Canada: 2022 surveillance highlights.

[3] Mbuagbaw L, Husbands W, Baidoobonso S, Lawson D, Aden M, Etowa J, Nelson L, Tharao W (2022). A cross-sectional investigation of HIV prevalence and risk factors among African, Caribbean and black people in Ontario: the A/C study. Can Commun Dis Rep.

[4] Centers for Disease Control and Prevention HIV Risk Reduction Tools.

[5] Ontario HIV Epidemiology and Surveillance Initiative (2021). HIV pre-exposure prophylaxis (PrEP) in Ontario, 2021.

[6] Tan DHS, Hull MW, Yoong D, Tremblay C, O'Byrne P, Thomas R, Kille J, Baril J-G, Cox J, Giguere P, Harris M, Hughes C, MacPherson P, O'Donnell S, Reimer J, Singh A, Barrett L, Bogoch I, Jollimore J, Lambert G, Lebouche B, Metz G, Rogers T, Shafran S, Biomedical HIV Prevention Working Group of the CIHR Canadian HIV Trials Network (2017). Canadian guideline on HIV pre-exposure prophylaxis and nonoccupational postexposure prophylaxis. CMAJ.

[7] Grant RM, Lama JR, Anderson PL, McMahan V, Liu AY, Vargas L, Goicochea P, Casapía M, Guanira-Carranza JV, Ramirez-Cardich ME, Montoya-Herrera O, Fernández T, Veloso VG, Buchbinder SP, Chariyalertsak S, Schechter M, Bekker L-G, Mayer KH, Kallás EG, Amico KR, Mulligan K, Bushman LR, Hance RJ, Ganoza C, Defechereux P, Postle B, Wang F, McConnell J J, Zheng J-H, Lee J, Rooney JF, Jaffe HS, Martinez AI, Burns DN, Glidden DV, iPrEx Study Team (2010). Preexposure chemoprophylaxis for HIV prevention in men who have sex with men. N Engl J Med.

[8] Zhabokritsky A, Nelson LE, Tharao W, Husbands W, Sa T, Zhang N, Thomas-Pavanel J, Baidoobonso S, Kaul R (2019). Barriers to HIV pre-exposure prophylaxis among African, Caribbean and Black men in Toronto, Canada. PLoS One.

[9] Djiadeu P, Yusuf A, Ongolo-Zogo C, Nguemo J, Odhiambo AJ, Mukandoli C, Lightfoot D, Mbuagbaw L, Nelson LE (2020). Barriers in accessing HIV care for francophone African, Caribbean and black people living with HIV in Canada: a scoping review. BMJ Open.

[10] Jackson GY, Darlington CK, Van Tieu H, Brawner BM, Flores DD, Bannon JA, Davis A, Frye V, Chittamuru D, Gugerty P, Koblin BA, Teitelman AM (2022). Women's views on communication with health care providers about pre-exposure prophylaxis (PrEP) for HIV prevention. Cult Health Sex.

[11] Mbuagbaw L, Hajizadeh A, Wang A, Mertz D, Lawson DO, Smieja M, Benoit AC, Alvarez E, Puchalski Ritchie L, Rachlis B, Logie C, Husbands W, Margolese S, Zani B, Thabane L (2020). Overview of systematic reviews on strategies to improve treatment initiation, adherence to antiretroviral therapy and retention in care for people living with HIV: part 1. BMJ Open.

[12] Ajiboye W, Nelson L, Odhiambo A, Yusuf A, Djiadeu P, Turner DA, Abubakari M, Pedersen C, Brown R, Ni Z, Guillaume G, Lofters A, Williams G (2022). Decision conflict and the decision support needs of HIV PrEP-eligible black patients in Toronto regarding the adoption of PrEP for HIV prevention. J Int Assoc Provid AIDS Care.

[13] Absalom D, Boyce T (2020). One Of These Things Aint Like The Other: Exploring the HIV prevention needs of Young Adult Black Same Gender Loving Men—A report prepared for the Gay Men?s Sexual Health Alliance.

[14] Stacey D, Légaré F, Lewis K, Barry MJ, Bennett CL, Eden KB, Holmes-Rovner M, Llewellyn-Thomas H, Lyddiatt A, Thomson R, Trevena L (2017). Decision aids for people facing health treatment or screening decisions. Cochrane Database Syst Rev.

[15] Garvelink MM, Boland L, Klein K, Nguyen DV, Menear M, Bekker HL, Eden KB, LeBlanc A, O'Connor AM, Stacey D, Légaré France (2019). Decisional Conflict Scale Findings among Patients and Surrogates Making Health Decisions: Part II of an Anniversary Review. Med Decis Making.

[16] Hoefel L, Lewis KB, O'Connor A, Stacey D (2020). 20th Anniversary Update of the Ottawa Decision Support Framework: Part 2 Subanalysis of a Systematic Review of Patient Decision Aids. Med Decis Making.

[17] Ottawa Patient Decision Aids research Group OHRI (2020). Inventory of personal decision aids.

[18] O'Connor AM, Jacobsen MJ Workbook on Developing and Evaluating Patient Decision Aids.

[19] O'Connor AM, Stacey D, Jacobsen M (2015). Ottawa Decision Support Framework (ODSF).

[20] O'Connor AM, Stacey D, Jacobsen M (2015). Ottawa Personal Decision Guide.

[21] Coulter A, Stilwell D, Kryworuchko J, Mullen PD, Ng CJ, van der Weijden T (2013). A systematic development process for patient decision aids. BMC Med Inform Decis Mak.

[22] Guillaume G, Ramos SR, M'Rabiu Abubakari G, Turner DE, Ajiboye W, Yusuf A, Djiadeu P, Odhiambo AJ, Pedersen C, Lofters A, Williams G, Nelson LE (2021). Barriers and facilitators to providing human immunodeficiency virus pre-exposure prophylaxis decision support to Black patients in Canada: a cross-sectional study. Int Health Trends & Persp.

[23] Mayer KH, Molina JM, Thompson MA, Anderson PL, Mounzer KC, De Wet JJ, DeJesus E, Jessen H, Grant RM, Ruane PJ, Wong P, Ebrahimi R, Zhong L, Mathias A, Callebaut C, Collins SE, Das M, McCallister S, Brainard DM, Brinson C, Clarke A, Coll P, Post FA, Hare CB (2020). Emtricitabine and tenofovir alafenamide vs emtricitabine and tenofovir disoproxil fumarate for HIV pre-exposure prophylaxis (DISCOVER): primary results from a randomised, double-blind, multicentre, active-controlled, phase 3, non-inferiority trial. Lancet.

[24] Ogbuagu O, Ruane PJ, Podzamczer D, Salazar LC, Henry K, Asmuth DM, Wohl D, Gilson R, Shao Y, Ebrahimi R, Cox S, Kintu A, Carter C, Das M, Baeten JM, Brainard DM, Whitlock G, Brunetta JM, Kronborg G, Spinner CD, DISCOVER study team (2021). Long-term safety and efficacy of emtricitabine and tenofovir alafenamide vs emtricitabine and tenofovir disoproxil fumarate for HIV-1 pre-exposure prophylaxis: week 96 results from a randomised, double-blind, placebo-controlled, phase 3 trial. Lancet HIV.

[25] O'Donnell S, Tan DHS, Hull MW (2019). New Canadian guideline provides evidence-based approach to non-occupational HIV prophylaxis. CJEM.

[26] Hughes C, Yoong D, Giguère Pierre, Hull M, Tan DHS (2019). Canadian guideline on HIV preexposure prophylaxis and nonoccupational postexposure prophylaxis for pharmacists. Can Pharm J (Ott).

[27] (2022). Differentiated and simplified pre-exposure prophylaxis for HIV prevention: update to WHO implementation guidance.

[28] Globerman J, Mitra S, Gogolishvili D, Rueda S, Schoffel L, Gangbar K, Shi Q, Rourke SB (2017). HIV/STI prevention interventions: A systematic review and meta-analysis. Open Med (Wars).

[29] US Public Health Service (2018). Preexposure prophylaxis for the prevention of HIV infection in the United States ? 2017 update: A clinical practice guideline.

[30] Jiang J, Yang X, Ye L, Zhou B, Ning C, Huang J, Liang B, Zhong X, Huang A, Tao R, Cao C, Chen H, Liang H (2014). Pre-exposure prophylaxis for the prevention of HIV infection in high risk populations: a meta-analysis of randomized controlled trials. PLoS One.

[31] Rapid Response Service (2018). Effectiveness of oral pre-exposure prophylaxis (PrEP) for HIV.

[32] Fagerlin A, Zikmund-Fisher BJ, Ubel PA (2011). Helping patients decide: ten steps to better risk communication. J Natl Cancer Inst.

[33] Ontario HIV Treatment Network PrEP Start.

[34] The Ottawa Hospital. A-Z inventory of decision aids. Patient decision aids. Link to the pre-exposure prophylaxis (PrEP) Decision Support Tool (DST).

[35] IPDAS 2005: Criteria for Judging the Quality of Patient Decision Aids.

[36] Elwyn G, Kreuwel I, Durand M, Sivell S, Joseph-Williams N, Evans R, Edwards A (2011). How to develop web-based decision support interventions for patients: a process map. Patient Educ Couns.

[37] Creswell JW, Clark VLP (2018). Designing and conducting mixed methods research (3rd ed.).

[38] The Ottawa Hospital Patient decision aids. A-Z inventory of decision aids.

